# Vaccination: the cornerstone of an efficient healthcare system

**DOI:** 10.3402/jmahp.v3.27041

**Published:** 2015-08-12

**Authors:** Vanessa Rémy, York Zöllner, Ulrike Heckmann

**Affiliations:** 1 Sanofi Pasteur MSD, Lyon, France; 2 Hamburg University of Applied Sciences, Hamburg, Germany; 3 Sanofi Pasteur MSD, Berlin, Germany

**Keywords:** vaccination, economic analysis, cost-effectiveness, cost-saving, public health

## Abstract

Vaccination has made an important contribution to the decreased incidence of numerous infectious diseases and associated mortality. In 2013, it was estimated that 103 million cases of childhood diseases in the United States had been prevented by the use of vaccines since 1924. These health effects translate into positive economic results, as vaccination can provide significant savings by avoiding the direct and indirect costs associated with treating the disease and possible long-term disability. A recent US study estimated that every dollar spent on childhood vaccination could save US$3 from a payer perspective and US$10 from a societal perspective. The first vaccines set a high standard from a public health ‘return on investment’ perspective, because they are highly cost-saving. Today, however, where only a few healthcare interventions are considered to be cost-saving, the challenge that decision-makers typically face is to identify such healthcare interventions that are deemed cost-effective, that is, provide extra benefit at a reasonable extra cost. Some of the newer vaccines provide a solution to some of today's important health issues, such as cervical cancers with human papillomavirus vaccines, or debilitating diseases with herpes zoster vaccines. These recent, more expensive vaccines have been shown to be cost-effective in several economic analyses. Overall, vaccination can still be regarded as one of the most cost-effective healthcare interventions.

During the 20th century, improved sanitation, nutrition, and the widespread use of antibiotics as well as vaccines have all contributed to the decreased incidence of numerous diseases and associated mortality. Vaccination was one of the public health measures that had the greatest impact on the reduction of the burden from infectious diseases and associated mortality, especially in children. It is estimated that, each year worldwide, vaccines prevent up to 3 million deaths (1, 2). In 1980, vaccination was responsible for the global eradication of smallpox for the first time in history. Vaccination has also led to the elimination of wild-type poliovirus in the Americas in 1990, in the Western Pacific Region in 2000, and in the European Region in 2002, and to the elimination of *Haemophilus influenza* type B (Hib) within a few years of introduction of conjugate Hib vaccines in many countries. Currently, there are more than 40 vaccines available for the prevention of 25 vaccine-preventable diseases (3). These health effects translate into positive economic results, and vaccination is commonly recognised as one of the most cost-effective public health investments (4, 5). However, most vaccines are considered to be underused; furthermore, they are probably undervalued (4). This article aims at examining the public health and economic impact of vaccination in industrialised countries, with a specific focus on Europe.

## Contribution from vaccination to public health

Vaccination has made a fundamental contribution to the prevention of numerous infectious diseases. Worldwide, it is estimated that vaccines prevent, annually, 5 million deaths caused by smallpox, 2.7 million cases of measles, 2 million cases of neonatal tetanus, 1 million cases of pertussis, 600,000 cases of paralytic poliomyelitis, and 300,000 cases of diphtheria (6).

**Fig. 1:**
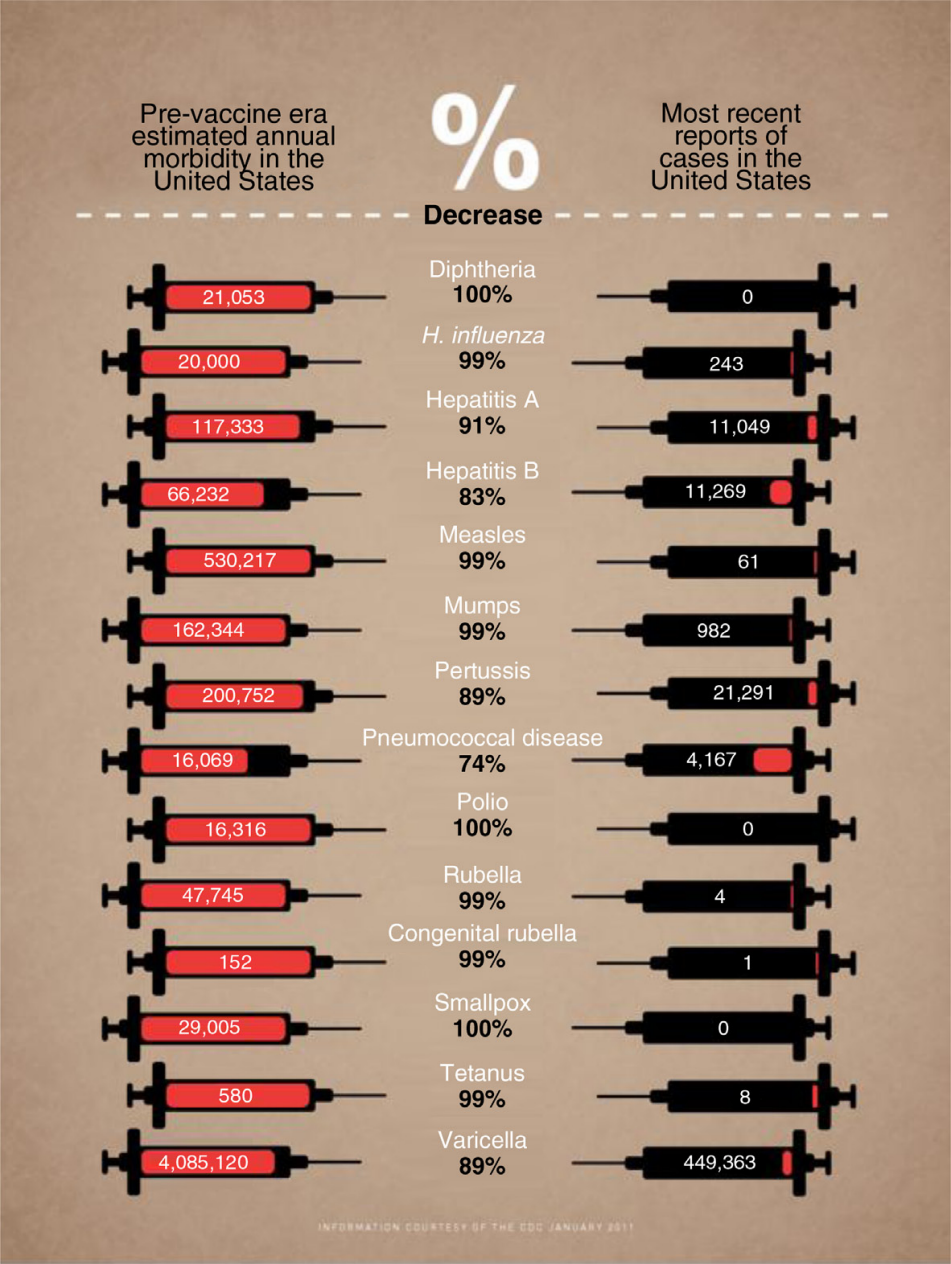
Comparison of the estimated annual morbidity in the United States in the pre- and post-vaccine eras (7).

In industrialised countries, several infectious diseases have been controlled and, in some cases, eliminated through routine vaccination. The generally high level of vaccination coverage has led to a dramatic decline in the reported incidence of many vaccine-preventable infectious diseases (Fig. 1) (7). A comparison between the period prior to the implementation of national vaccination recommendations in the United States and 2006 showed a greater than 99% decline in the number of cases of diphtheria (100%), measles (99.9%), paralytic poliomyelitis (100%), and rubella (99.9%). A greater than 92% decline in cases and a 99% or greater decline in deaths were shown for mumps, pertussis, and tetanus (8, 9). In 2013, it was estimated that 103 million cases of childhood disease in the United States had been prevented by the use of vaccines since 1924, of which 26 million cases in the past decade alone (10). A similar trend has been observed in Europe (Table 1) (9, 11). In France, diphtheria, tetanus and polio, BCG (tuberculosis), and pertussis vaccines were estimated to be responsible for saving more than 400,000 years of life (4).

**Table 1:** Number of reported cases of vaccine-preventable diseases in the European region based on data from the WHO vaccine-preventable disease monitoring system

	1980	2000	2011	2012	2013
Diphtheria	608	1,585	33	32	32
Measles	851,849	37,421	37,073	26,982	25,375
Mumps	No data	243,344	27,448	38,141	35,075
Pertussis	90,546	53,675	34,432	56,941	27,824
Polio	549	0	0	0	0
Rubella	No data	621,039	9,672	30,509	39,614
Rubella (CRS)	No data	48	7	60	50
Tetanus	1,715	412	197	194	93

From Refs (9, 11). CRS: congenital rubella syndrome; Full database available in Ref. (11).

Another example is *H. influenzae* type b (Hib) invasive disease, which was the leading cause of childhood meningitis and was associated with high death rates and sequelae. Before a Hib vaccine was available, an estimated 445,000 cases of invasive Hib disease occurred in children under 5 years of age globally, each year, 115,000 of which resulted in death. The incidence of Hib meningitis in Europe has been reduced by more than 90% in less than 10 years because of vaccination (4).

## Vaccination represents a valuable investment in health with positive economic return

Whether the benefits are reported in terms of avoided deaths, life-years saved, disability-adjusted life years (DALYs) avoided or quality adjusted life years (QALYs) gained, vaccination is universally considered to provide important public health benefits (12). These health effects translate into positive economic outcomes. Vaccination can provide significant savings by avoiding the health costs associated with treating diseases. Table 2 summarises the results of a US study estimating the direct and indirect (i.e., loss of productivity) costs savings for several vaccine-preventable diseases (13).

**Table 2:** Direct and indirect savings from vaccination

Disease	Comparative savings	Direct or indirect savings (US$)
Smallpox^a^	NA	300 million in direct costs per year
Polio^b^	NA	13.6 billion in total savings world wide by 2040 700 million in the United States between 1991 and 2000
Measles	Treating one child with measles costs 23 times the cost of vaccinating one child against measles	10 per disability-adjusted life-year (DALY)
Cholera	NA	770 million lost in seafood exports in Peru, 1991
Malaria	NA	100 billion GDP lost annually in sub-Saharan Africa
MMR	For every US$ spent on MMR vaccine, more than US$21 is saved in direct medical care costs	100 million in direct medical costs from 1989 to 1991 for measles outbreaks
DTaP	For every US$ spent on DTaP vaccine, US$24 is saved	23.6 billion in direct and indirect costs without DTaP vaccines
Hib	For every US$ spent on Hib vaccine, more than US$2 is saved	5 billion in direct costs and 12 billion in indirect costs incurred in the United States

From Ref. (13). NA: not available; MMR: measles–mumps–rubella; DTaP: diphtheria–tetanus–acellular pertussis; Hib: *H. influenza*e type b.

a Based on eradication of smallpox in 1977;

b based on eradication of polio by 2005; calculation details available in Ref. (13).

The economic impact of vaccination programmes has been evaluated through different economic indicators, such as benefit–cost ratio (BCR=total discounted benefits divided by total discounted programme costs, if >1: benefits outweigh the costs), the net benefit (total discounted benefits minus total discounted costs) and return on investment (ROI=net benefit divided by costs, if >0: benefits exceed the costs), as illustrated in the following examples. Current childhood vaccines against diphtheria, tetanus, pertussis, Hib, polio, measles, mumps, rubella, and hepatitis B, when considered together, were estimated to have a BCR of more than 5:1 for direct costs and 17:1 for societal costs (14). A recent US study confirmed the pattern of this finding, estimating that every dollar spent on childhood vaccination saves US$3 from a payer perspective (i.e., direct costs) and US$10 from a societal perspective (i.e., direct and indirect costs; Table 3) (15). In the United States, the diphtheria, tetanus, and pertussis (DTP) vaccine has resulted in direct and indirect cost savings of US$23.6 billion, with a corresponding BCR of 27:1 (16). In another US study, it was estimated that the net benefit for 60 years of investment in polio vaccine was six times higher (approximately US$180 billion) than the total investment over the same period (approximately US$36.4 billion) (17). A European review, taking the UK as an example, demonstrated that for every euro spent on targeted influenza vaccination for the elderly, €1.35 savings were generated in terms of reduced medical spending elsewhere (18) in the healthcare system. In Europe, an Italian study reported that universal hepatitis B childhood vaccination would have a positive economic impact 20 years after its implementation (19). The ROI was estimated to be almost 1 from the National Health Service perspective, and the BCR slightly less than 1 from the societal perspective, considering only the first 20 years after the start of the programme. With a longer term horizon, both the ROI and BCR values were estimated to be positive (2.78 and 2.47, respectively). The hepatitis B vaccination programme in Italy is a clear example of the massive impact that universal vaccination can have on the medium-to-long-term, when healthcare authorities are wise enough to invest in prevention (19).

**Table 3:** Summary of an economic evaluation of the routine childhood vaccination programme in the US in 2009

Childhood vaccination programme	Payer perspective	Societal perspective
Costs saved	20.3	76.4
Costs of routine immunization programme	6.7	7.5
Net cost savings	13.5	68.8
Benefit–cost ratio	3.0	10.2

Costs are given as 2009 billion US$. *Note*: Calculations based on population-based vaccination coverage, published vaccine efficacies, historical data on disease incidence before vaccination, and disease incidence reported during 2005–2009. Programme costs included vaccine, administration, vaccine-associated adverse events, and parent travel and work time lost. Three percent annual discount rate (15).

Investments in infectious disease eradication have also proven highly valuable. For example, the World Health Organisation invested more than US$300 million over 11 years in the Intensified Smallpox Eradication Programme (1967–1979). This investment has paid back many times by saving human lives and by the elimination of downstream costs for vaccines, treatment, and international surveillance activities. The annual savings from smallpox eradication are estimated to be more than US$2 billion each year; these savings have been used for other pressing health issues (4). A similar trend could be expected if polio eradication were achieved: ‘The world as a whole is expected to save US$1.5 billion a year once vaccination is discontinued, of which the United States would save about US$230 million’ (20).

## Modern vaccines: continued good value for money

Vaccination is often considered as the most cost-effective public health intervention after clean water (4, 21). The first vaccines set a high standard because they were cost-saving, i.e., the money invested in vaccination programmes was completely offset by the treatment costs avoided. These vaccines were introduced in an environment of poorer quality of population health and sanitary conditions, with a very high incidence and morbidity of infectious diseases. Today's new vaccines are available in a better health environment and represent a solution to our health issues today, such as cancers or debilitating diseases. Compared with the original vaccines, these new vaccines are more costly, partly as a result of their more advanced and complex, patent-protected technologies, such as recombination techniques, carrier proteins, and adjuvants (22). In addition, recent analyses suggest that increased regulatory oversight is another factor driving up the price of new vaccines (22, 23). However, economic analyses have reported that, despite their higher costs, new vaccines have been found to be cost-effective (according to commonly used thresholds in Europe ranging from €20,000 to €50,000/QALY), meaning that they provide good health value at a cost deemed reasonable, according to payers’ willingness to pay (24). For example, a systematic review analysed 15 published economic evaluations on the human papillomavirus (HPV) vaccine performed in Europe, of which 10 were industry-sponsored, while 5 were not (25). Interestingly, the authors reported that nine sponsored studies as well as the five non-sponsored studies were favourable to HPV vaccination cost-effectiveness, while one of the 10 industry-sponsored studies was not (25). In another systematic review of the cost-effectiveness of zoster vaccination, all but one of the studies included in the review concluded that most vaccination scenarios were cost-effective (26). However, comparisons between cost-effectiveness studies may be difficult because of variability and uncertainty around model assumptions (i.e., perspective, model design, time horizon, comparators, etc.) or input data applied between studies and countries. For example, a systematic review reported that rotavirus vaccination was found to be cost-effective in developing countries but that conclusions varied between studies in developed countries (27). Rotavirus vaccination was likely to be cost-effective under some scenarios, such as inclusion of herd protection and adoption of a societal perspective, demonstrating the need to thoroughly evaluate studies’ comparability before drawing any conclusion.

## Conclusion

Vaccination has made a fundamental contribution to the decreased incidence of numerous infectious diseases and associated mortality. These health effects translate into positive economic outcomes for healthcare systems and to society as a whole. Vaccines are generally regarded as one of the most cost-effective public health measures available. However, they are often undervalued and/or underused, though for different reasons: undervalued, paradoxically, in some parts of the world where increased vaccination coverage could provide significant benefit; underused, in other parts of the world where the high standards of health and healthcare seem to be have led to the achieved vaccine-borne benefits being taken for granted, in these societies at risk of complacency.

The under-utilisation of vaccines in industrialised countries could be seen as vaccination being a victim of its own success, leading people to underestimate the seriousness of vaccine-preventable diseases and the benefits of vaccination, and, instead, to have concerns regarding the side effects of vaccines. As for any biological or medicinal product, adverse reactions due to vaccines, although extremely rare, exist. For example, the risk a child will have a severe reaction after receiving the MMR (Measles, Mumps, and Rubella) or DTaP vaccine is less than 1 in 1,000,000 (28). Additionally, even if vaccination is one of the most cited examples of positive externalities, through herd immunity and reduced transmission of the disease, it may also have potentially negative epidemiological effects such as serotype replacement or shift of disease to older populations. These potential effects should be closely monitored and weighed against the benefits of protecting from severe vaccine-preventable diseases to conclude on the benefit/risk profile of a particular vaccination programme.

In parallel, the ability to reduce the global disease burden with vaccines continues to grow, as new vaccines are developed to prevent other diseases and policy-makers must decide where and how scarce resources are best allocated. Product- and programme-specific attributes such as safety, efficacy, feasibility, and cost-effectiveness play an important role in the basic health system objectives of efficiency, equity, and sustainability. The earlier vaccines set a high standard because they were not only cost-effective, but often even cost-saving, turning decision-making into a relatively straightforward task (requiring only strictly rational behaviour). However, it seems too narrow today to expect that vaccines should be cost-saving (i.e., ‘pay for themselves’), especially in the short-term, since this would suggest that investing in preventive measures is not worthwhile. The new generation of vaccines, despite not always being cost-saving, has been shown to be cost-effective by many research teams, in a multitude of scenarios.

Ultimately, it is the global society and future generations that benefit when all countries make the effort to protect their populations from vaccine-preventable diseases. As such, vaccination programmes need adequate support and recognition of their value for an efficient and timely implementation and realisation of their full potential (23).
